# Interaction between nitric oxide and renal α_1_-adrenoreceptors mediated vasoconstriction in rats with left ventricular hypertrophyin *Wistar Kyoto* rats

**DOI:** 10.1371/journal.pone.0189386

**Published:** 2018-02-15

**Authors:** Ashfaq Ahmad, Munavvar A. Sattar, Maleeha Azam, Safia A. Khan, Owais Bhatt, Edward J. Johns

**Affiliations:** 1 School of Pharmaceutical Sciences, Universiti Sains Malaysia, Penang, Malaysia; 2 Department of Pharmacology and Toxicology, Virginia Commonwealth University, Richmond, Virginia, United States of America; 3 Translational Genomics Lab, Department of Biosciences, COMSATS Institute of Information Technology, Islamabad, Pakistan; 4 Department of Physiology, University College Cork, Cork, Ireland; Max Delbruck Centrum fur Molekulare Medizin Berlin Buch, GERMANY

## Abstract

Left ventricular hypertrophy (LVH) is associated with decreased responsiveness of renal α_1_-adrenoreceptors subtypes to adrenergic agonists. Nitric oxide donors are known to have antihypertrophic effects however their impact on responsiveness of renal α_1_-adrenoreceptors subtypes is unknown. This study investigated the impact of nitric oxide (NO) and its potential interaction with the responsiveness of renal α_1_-adrenoreceptors subtypes to adrenergic stimulation in rats with left ventricular hypertrophy (LVH). This study also explored the impact of NO donor on CSE expression in normal and LVH kidney. LVH was induced using isoprenaline and caffeine in drinking water for 2 weeks while NO donor (L-arginine, 1.25g/Lin drinking water) was given for 5 weeks. Intrarenal noradrenaline, phenylephrine and methoxamine responses were determined in the absence and presence of selective α_1_-adrenoceptor antagonists, 5- methylurapidil (5-MeU), chloroethylclonidine (CeC) and BMY 7378. Renal cortical endothelial nitric oxide synthase mRNA was upregulated 7 fold while that of cystathione γ lyase was unaltered in the NO treated LVH rats (LVH-NO) group compared to LVH group. The responsiveness of renal α_1A_, α_1B_ and α_1D_-adrenoceptors in the low dose and high dose phases of 5-MeU, CEC and BMY7378 to adrenergic agonists was increased along with cGMP in the kidney of LVH-NO group. These findings suggest that exogenous NO precursor up-regulated the renal eNOS/NO/cGMP pathway in LVH rats and resulted in augmented α_1A_, α_1B_ and α_1D_ adrenoreceptors responsiveness to the adrenergic agonists. There is a positive interaction between H_2_S and NO production in normal animals but this interaction appears absent in LVH animals.

## Introduction

Left ventricular hypertrophy (LVH) is characterized by overstimulation of the heart due to hyperactivity of the sympathetic nervous system and both circulating noradrenaline and mean discharge frequency in peripheral sympathetic nerves have been reported elevated in hypertensive LVH patients [1].At an experimental level, renal sympathetic nerve activity was found to be elevated in rats with essential hypertension and LVH compared to the control *Wistar Kyoto* rats [2]. This sympatho-activation is associated with vascular dysfunction and impairment of α_1_-adrenoceptor-mediated renal vasoconstriction [3].This attenuation of α_1_-adrenoceptor-mediated renal vasoconstrictor responsiveness to adrenergic agonists in states of hypertension and renal failure has been studied previously [4]. Moreover, a decrease in responsiveness of α_1D_–adrenoreceptors to adrenergic agonists when administered exogenously has been reported LVH [5]. However, the question of the role of NO on the responsiveness of α_1_-adrenoceptors in LVH remains unanswered.

Higher levels of noradrenaline (NA) and angiotensin II (Ang II) in the plasma have been found in rat models of LVH induced using isoprenaline and caffeine [5–7] At the level of renal vasculature, catecholamines are released at the sympathetic nerve neuro-effector junctions and activate the G-protein operated adrenoreceptors which increase cytosolic Ca^2+^ concentration to vascular smooth muscle contractions [8]. Pharmacological and cloning studies have reported three subtypes of α_1_-adrenoceptors, α_1A_, α_1B_ and α_1D_ [9]. These α_1_-adrenoceptors are operated by G-protein coupled receptor 2^nd^ messenger signalling pathway [9]. Increased vasoconstriction due to elevated NA and Ang II can be suppressed as a result of an up-regulation of the NO-cGMP pathway which is responsible for inhibition of L-type Ca^2+^ channels [10] which induce a vasodilation.

Nitric oxide derived from endothelial nitric oxide synthase (eNOS) is important in maintaining and determining normal renal hemodynamic and tubular reabsorptive function [11, 12]. Nitric oxide has been reported to reduce renal ischemia reperfusion injury [13] both directly and indirectly [14].There is evidence demonstrating that NO exerts a tonic role in the medullary circulation [15] where it seems to have a higher concentration than in the cortex [16]. Earlier studies have shown that intravenous infusion of endothelial cells (eNOS) in ischemic kidney provides dramatic renoprotection by lowering plasma creatinine [17, 18]. We reported recently that the down regulation of the eNOS/NO pathway was associated with a decrease in responsiveness of α_1A_–adrenoreceptors to adrenergic agonists in the kidney of LVH rats [19]. Decreased responsiveness of α_1_-adrenoreceptors has been reported in many pathological conditions such as hypertension and renal failure [4], in fructose fed rats and in LVH [5]. Although these studies provide an elegant insight as to the renal consequences of reduced responsiveness of α_1_-adrenoceptors to adrenergic agonists, no study has been conducted to determine the impact of an increase in the responsiveness of α_1_-adrenoreceptors in different pathological conditions.

Various studies have shown that production of both H_2_S and NO are interdependent [20–24] in regulating vascular tone. Literature showed that H_2_S yield NO production in smooth muscles [25,26] while it has also been reported that NO enhanced the up regulation of H_2_S production as reflected by plasma concentrations [27, 28].

The potential interaction between NO and α_1_–adrenoceptor subtypes of normal or LVH animals in regulating renal hemodynamic has not been investigated to date. Collectively, the evidence available regarding NO plus our recent findings of an interaction between eNOS/NO α_1_–adrenorecptors subtypes in the kidney of LVH rats raises a number of questions. The hypothesis to be explored is as follows: firstly, that upregulation of the eNOS/NO/cGMP pathway will increase the responsiveness of the renal vascular α_1_–adrenoreceptor subtypes to adrenergic agonists in LVH rats; secondly, that up regulation of eNOS/NO in kidney will improve renal cortical blood perfusion in LVH; thirdly, that chronic administration of L-arginine (an NO donor) will suppress the CSE/H_2_S pathway in the kidney of LVH rats.

## Materials and methods

### Animals and induction of LVH

All the procedures of current study were approved by the Animal Research and Service Centre (ARASC) USM with approval no./2012/ (76) (364) and all the methods were performed by the guidelines and procedures as approved by ARASC. 84 male Wistar *Kyoto* (WKY) rats (200±10g) were recruited from the animal house of Universiti Sains Malaysia and kept in standard animal facility provided by School of Pharmaceutical Sciences, USM with free access to food and water. All animals were divided into three main groups; one for renal functional studies, a second group for CSE and eNOS mRNA evaluation and a third group for the measurement of nitric oxide synthase (NOS) protein expression. The main group for renal hemodynamic functional examination of α_1_-adrenoceptors subtypes consisted of 12 subgroups groups. These groups were named according to antagonists used in that group. Renal functional study group consists of:

(1) Control-5MeU; (2) Control-CEC; (3) Control-BMY. LVH groups consisted of: (4) LVH-5MeU; (5) LVH-CEC; (6) LVH-BMY. Control groups treated with NO consisted of: (7) Control-NO+5MeU; (8) Control-NO+ CEC; (9) Control-NO+BMY.LVH groups treated with NO consisted of: (10) LVH-NO+5MeU: (11) LVH-NO+CEC; (12) LVH-NO+BMY (n = 6). Similarly, molecular study for quantification of CSE and eNOS mRNA expression consisted of 4 groups: Control, LVH, Control-NO and LVH-NO whereby the cortex part of left kidneys were taken for quantification of CSE and eNOS mRNAs expression. A third group, Control (Control-L-NIO) and a LVH group (LVH-L-NIO),which received L-N5-(1-iminoethyl)-ornithine), (10mg/kg I.P.) 15 minutes before the acute experiment [29] and NOS activity was compared to a control group (control-L-NIO).

LVH was induced by a modification of an earlier model [30] using isoprenaline (5mg/kg s.c) and caffeine as recently reported [5].Control-NO and LVH-NO group rats received L-arginine (1.25 g/L in the drinking water) was used as a donor of NO for 5 weeks as reported previously [31]. Control rats received i.ps injection of 0.9% NaCl.

### Molecular expression of *CSE* and *eNOS mRNAs* in the cortex of the kidney

Molecular expression study was performed following the procedure reported earlier [19]. Conversion of RNA to cDNA was performed by using a High Capacity RNA-to-cDNA kit (Applied Biosystems™, USA) according to the manufacturer’s instruction.

Different TaqMan primers and probes were used for gene which have following accession numbers; CSE gene (Gen Bank accession No. NM_017074.1 and Rn00567128_m1) [32]; eNOS genes (Gen Bank accession No. NM_021838.2 and Rn02132634_s1) [33, 34] and for the β-actingene (Gen Bank accession No. NM_031144.2 and Rn00667869_m1) were derived from TaqMan^**®**^-Gene Expression assays (Applied Biosystems, USA) [35, 36].TaqMan® Gene Expression assays were obtained and the procedure was followed according to the instructions of the manufacturer (Applied Biosystems™, USA).

Quantitative RT-PCR reactions were carried out on cortex of the left kidney. Amplification of the housekeeping enzyme (internal control) Beta actin allowed sample loading and normalization to be determined. The relative quantification of the target genes CSE, eNOS and internal control beta actin used the comparative C_T_ (threshold cycle) method with arithmetic formula (2^-ΔΔCT^) [37].

### *NOS* enzyme activity in kidney

NOS enzyme activity was done as reported in earlier studies [38, 39]. Enzyme activity was expressed as citrulline production in femtomol per milligram of protein per minute.

### Measurement of nitric oxide concentration in the plasma and kidney

The plasma and tissue concentration of nitric oxide was measured using kits as directed by manufacturer (NJJC Bio Inc., Nanjing, China) while protein quantity was measured using an early reported method [39, 40]. Blood was collected from the rat and centrifuged at 5000g for 10 minutes to collect plasma for analysis of nitric oxide.

### Measurement of hydrogen sulphide concentration in the plasma

The plasma concentration of H_2_S was measured as reported previously [41, 42].

### Measurement of cGMP levels in the kidney

The method used followed the instructions provided by manufacture of the cGMP Direct Immunoassay Kit (Abcam). However, procedure involves sample preparation, construction of standard curve, followed by acylation and then quantification of cGMP by measuring the optical density at 450nm.

### Acute experiment for renal vasoconstrictor responses

*In vivo* renal vasoconstrictor responses studies were performed as previously reported [43]. Animals were anaesthetized by intraperitoneal pentobarbitone sodium (60mg/kg, Nembutal**®**, CEVA, France) injections. Tracheotomy was done by inserting tubing in the trachea to facilitate breathing followed by cannulation of jugular vein and carotid artery for vehicle infusion and continuous MAP monitoring respectively. Furthermore, carotid artery cannula was connected to a pressure transducer (model P23 ID Gould, Statham Instruments, UK) which was further attached to a PowerLab data acquisition system (PowerLab, ADInstruments, Australia). A mid-line abdominal incision was made to expose the aorta and left kidney and a laser Doppler probe (ADInstruments, Australia) was placed on the cortical surface of the left kidney to measure renal cortical blood perfusion (RCBP). In order to facilitate the close infusion of adrenergic agonist noradrenaline (NA), phenylephrine (PE) and methoxamine (ME) close to the face of renal artery, left iliac artery was cannulated and cannula was pushed at the required level designed by study [44, 45]. Animals were allowed to stabilize for 1 hour before commencing acute vasoconstrictor studies.

### Renal vasoconstrictor responses

Different doses of NA, PE and ME were administered intrarenally in ascending and descending order as described below; NA at 25, 50, 100 and 200ng; PE at 0.25, 0.5,1 and 2μg; ME at 1, 2, 3 and 4 μg. These drugs were prepared in saline (0.9g of NaCl/L of water) freshly every day and stored in 4°C [42, 46]. A wash out time of 10 min was given to each dose administered to ensure washout of agonists [47, 48]. Overall acute experiment consisted of three phases, a saline or non-drug phase, a low dose antagonist phase and a high dose antagonist phase. In the saline phase, saline was infused intrarenally at a rate of 6ml/kg/h during which adrenergic agonists were infused in ascending and descending order. In the low and high dose phases, 5-MeU was administered close to renal artery at bolus dose of 5μg/kg and plus infusion of 1/4^th^ of the bolus dose as a continuous infusion (1.5μg/kg/h) to study α_1A_adrenoreceptors while during the high dose phase 5-MeU was administered as a 10μg/kg bolus dose followed by a continuous infusion of 2.5μg/kg/h. Chloroethylclonidine was administered in low dose (5mg/kg as bolus dose) and high dose (10 mg/kg as bolus) in kidney [49]. BMY 7378 was infused intrarenally at 100 and 200mg/kg plus 1/4^th^ the dose as a continuous infusion, for the low and high dose phases, respectively, during which adrenergic agonists were administered [4].

### Histopathology of kidney tissues using haematoxylin and eosin staining

At the end of experiment right kidneys were removed and tissues for all four groups were subjected to the histopathological process of staining as reported [39, 50].

### Histopathology study of the kidney using picrosirus red stain kit

The same preparative procedure given above was repeated for staining with Picrosirus red (Polyscience, Inc. Germany) as reported [39] and directed by manufacturer.

### Preparation of agonists and antagonists

5-methylurapidil (RBI, Natick, MA, USA) is a selective blocker of α_1A_adrenoreceptors [51], chloroethylclonidine (RBI, Natick, MA, USA) a selective blocker of α_1B_adrenoreceptors [52] and BMY 7378 (8-(2-(4-(2-methoxyphenyl)-1-piperazinyl)ethyl)-8-azaspiro(4,5) decane-7,9-dione dihydrochloride; RBI) a selective blocker of α_1D_ adrenoreceptors [53], were prepared in saline and kept frozen as stock solutions.

## Statistical analysis

The renal vasoconstrictor response to each agonist was taken as the mean of ascending and descending responses due to four doses which are shown as line graphs as shown in supplementary data. The comparison between the groups considered the overall response calculated as the mean of the % of drop in renal cortical blood profusion pressure. All data was presented as mean ± S.E.M. The renal vasoconstrictor data were subjected to a one-way ANOVA followed by a Bonferroni *post hoc* test using GraphPad Prism (GraphPad SoftwareInc., CA, USA) with significance taken at P< 0.05. The gene expression data was analysed using the comparative method (ΔΔC_T_ method) and using the StepOne^™^ Software (Version 2.1, Applied Biosystem, USA).

## Results

### Molecular expression of renal cortical *CSE* and *eNOS*

**Fig 1:**
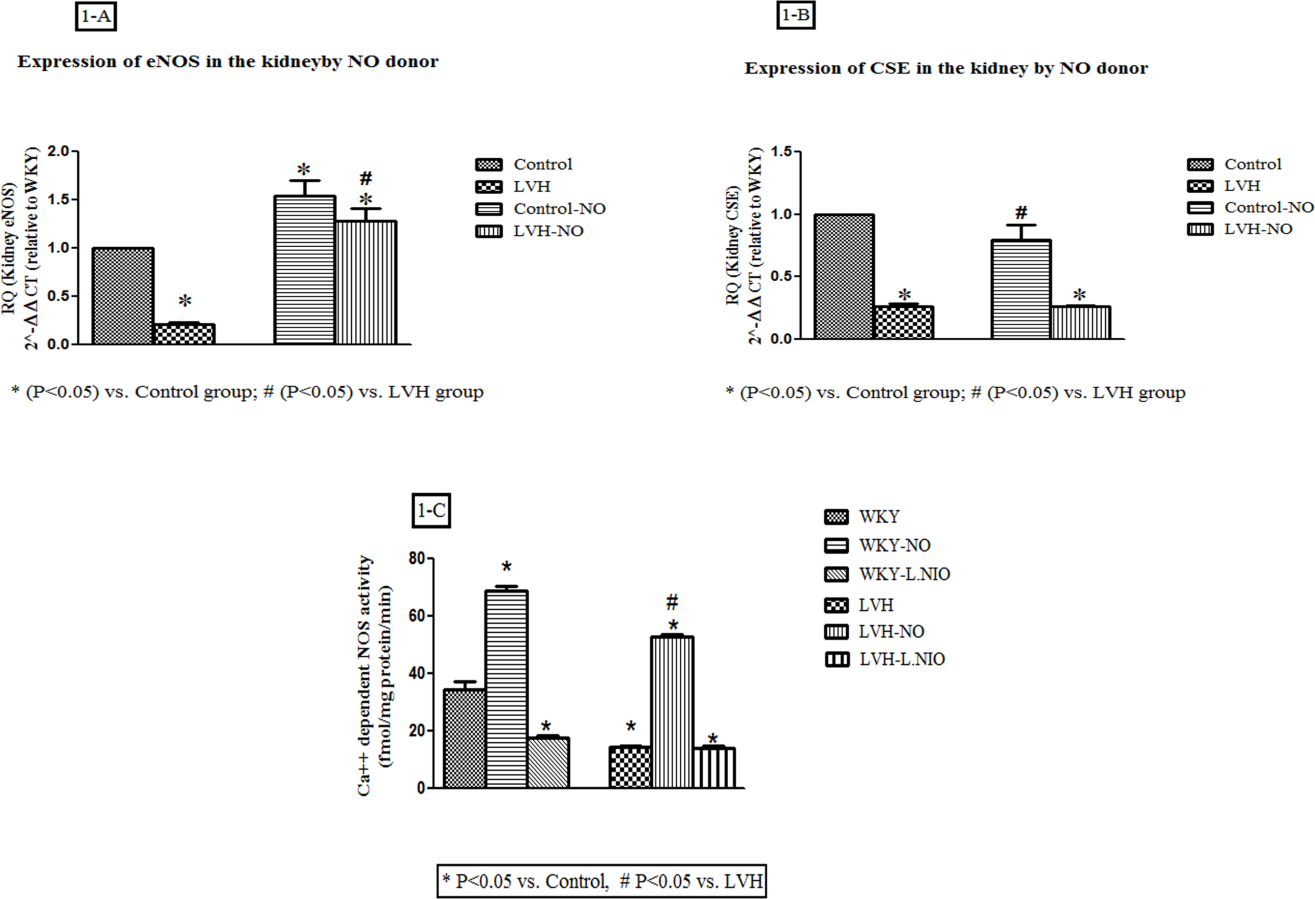
A, B, C) showing the molecular expression of CSE and eNOS mRNAs and NOS activity in the cortex of kidney of the Control, LVH, Control-NO and LVH-NO rats. Data is shown as ± SEM while significance is taken as p<0.05.

Induction of LVH resulted in a 79% down regulation of eNOS mRNA in the renal cortex compared to that in the control rats. Treatment of LVH with L-arginine resulted in the 510% increase in eNOS mRNA when compared to LVH groups as shown in Fig 1A

Induction of LVH resulted in a 73% down regulation of CSE mRNA in the renal cortex compared to its expression in the control rats. Treatment with L-arginine in the Control rats increased CSE mRNA by 204% compared to the untreated counterpart but had no impact on the expression levels in the LVH rats as shown in Fig 1B.

### NOS enzyme activity in kidney

Ca^2+^-dependent NOS activity was reduced significantly (all P<0.05) in the kidney of LVH when compared to the control group while exogenous administration of L-arginine in LVH significantly increased (all P<0.05) NOS activity when compared to LVH as shown in Fig 1C.

### Renal and plasma nitric oxide concentrations

**Fig 2:**
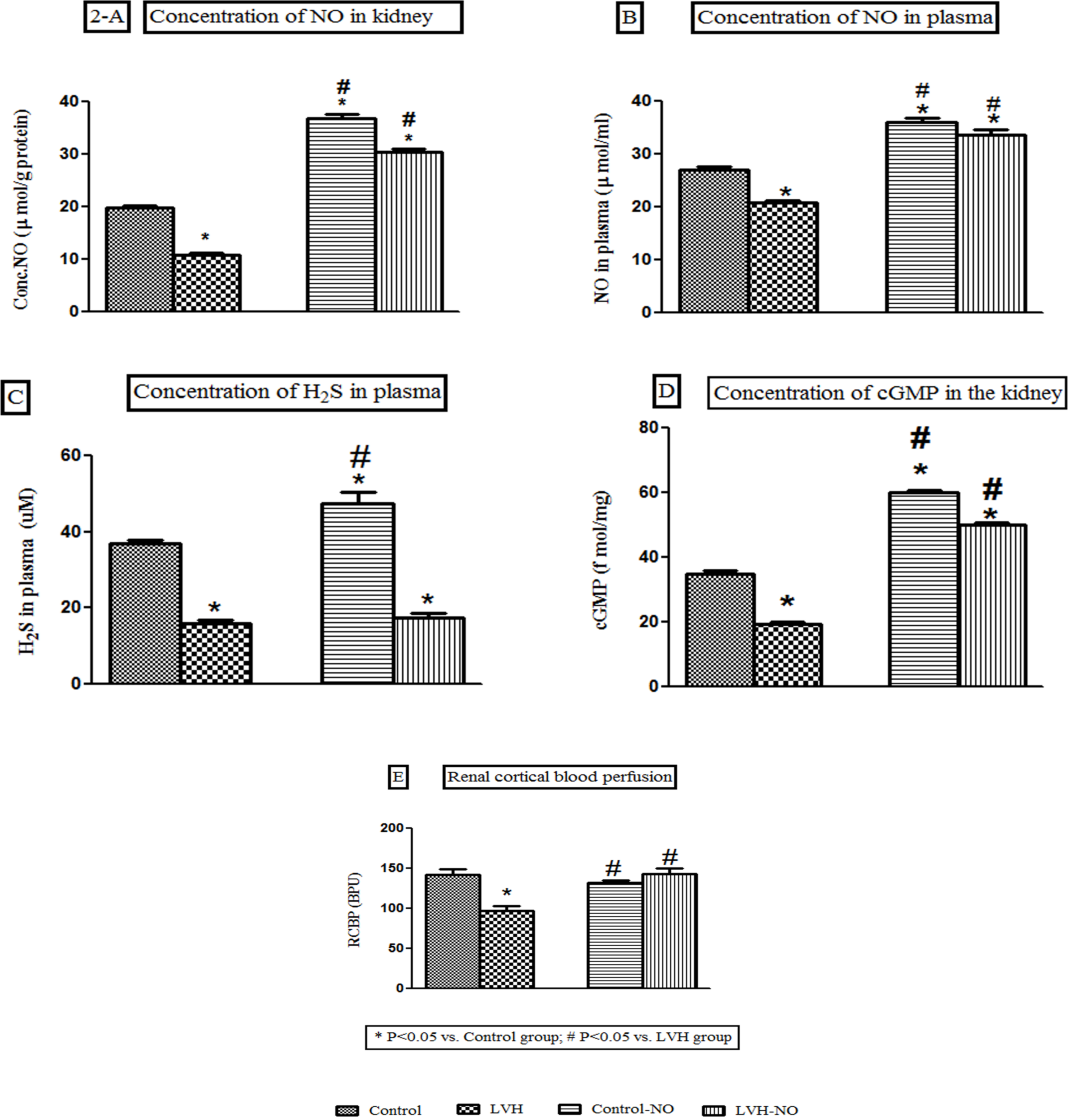
Showing the concentration of nitric oxide in the kidney (A), nitric oxide in plasma(B), H_2_S in plasma (C), cGMP in the kidney (D) and renal cortical blood perfusion of Control, LVH, Control-NO and LVH-NO rats. Data is shown as ± SEM while significance is taken as p<0.05. * (P<0.05) vs. Control group; # (P<0.05) vs. LVH group.

Induction of LVH resulted in 45% decrease in renal NO concentrations compared to the control rats. Treatment with L-arginine resulted in a 236% and 173% increase in renal NO concentrations in the Control-NO and LVH-NO groups, respectively as shown in Fig 2A.

Induction of LVH caused a 29% decrease in plasma NO concentration compared to that in the control rats. Treatment with L-arginine resulted in increased plasma NO concentration of 71% and 52% in the Control-NO and LVH-NO groups, respectively as shown in Fig 2B.

### Plasma hydrogen sulphide concentrations

The plasma concentration of H_2_S was significantly (P<0.05) lower in the LVH group compared to the Control group (16±1 vs.37±1μM) and was unchanged following treatment with L-arginine compared to LVH (18± vs. 16±1μM) as shown in Fig 2C.

### Renal cGMP concentrations

Induction of LVH decreased renal concentrations of cGMP by 84% compared to the control rats. Treatment with L-arginine increased renal cGMP concentrations by 216% and 163% in the Control-NO and LVH-NO groups, respectively as shown in Fig 2D.

### Renal cortical blood perfusion

RCBP was 46% lower (P<0.05) in the LVH compared to the Control rats. Treatment with L-arginine resulted in a higher RCBP in Control-NO and LVH-NO of some 36% and 47%, respectively as shown in Fig 2E.

### Renal vasoconstrictor responses of α_1A_–adrenorecptors to adrenergic agonists

#### Noradrenaline

**Fig 3:**
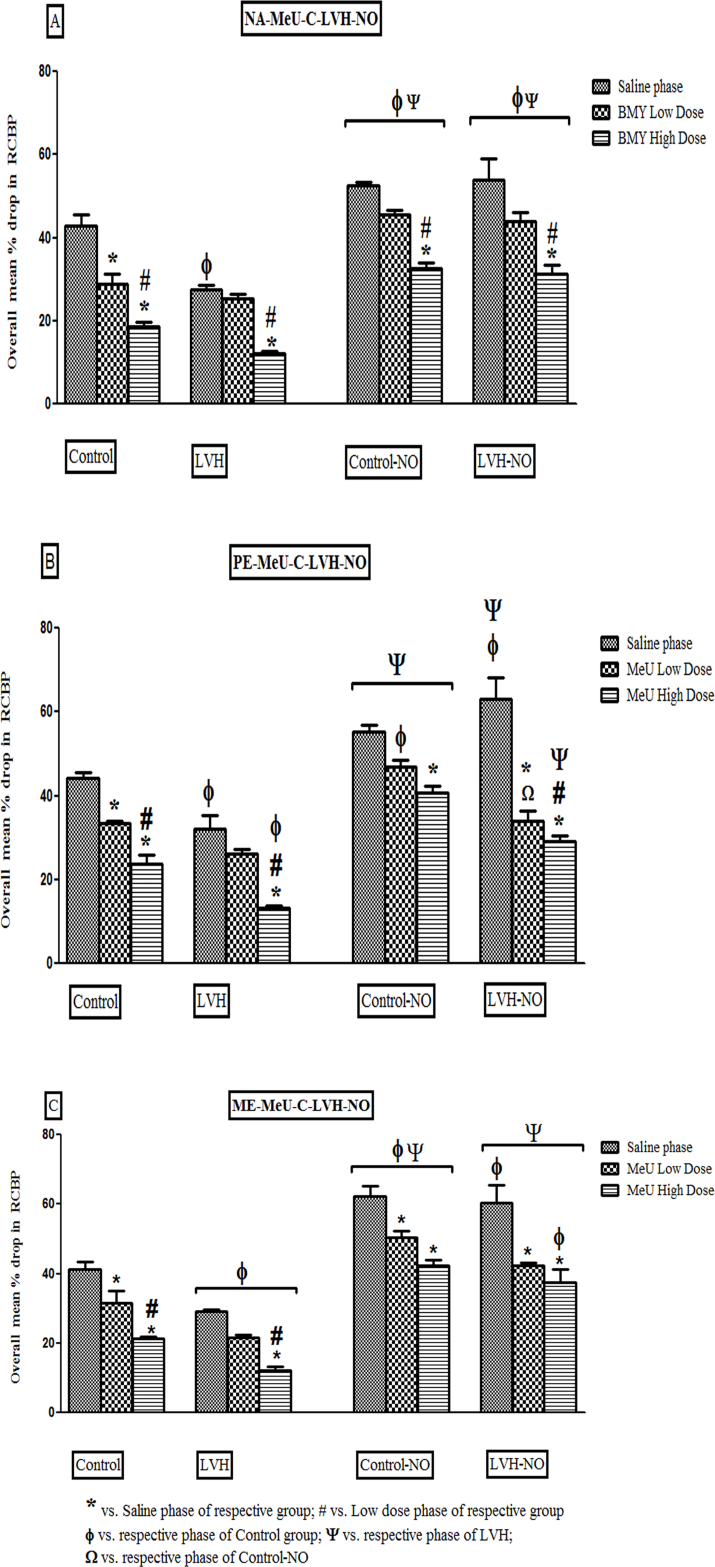
Showing the renal vasoconstrictor responses of α_1A_adrenoreceptors to NA (3A), PE (3B) and ME (3C) in the kidney of Control, LVH, Control-NO and LVH-NO rats. Data is shown as ± SEM while significance is taken as p<0.05.

The reductions in renal cortical blood perfusion (RCBP) in the LVH group were 33% lower in the saline and 37% during the high dose phases of the antagonists (P<0.05) when compared to same phases in the Control groups of rats. Treatment of LVH with L-arginine resulted in augmented responses to the α_1A_–adrenoreceptor agonist NA, by 93% in the saline phase, 76% in low dose 5-MeU phase and 158% in the high dose 5-MeU phase when compared to respective phases in the LVH group as shown in Fig 3A (S1 Fig).

#### Phenylephrine

There was a reduced responsiveness to PE in the LVH group, of 27% in saline and 46% in the high dose phase of 5-MeU (P<0.05) while there was no significant difference observed during the low dose phase of antagonist when compared to same phases of Control groups of rats. Treatment of LVH with L-arginine augmented the RCBP responses to PE by 97% in the saline and 123% in high dose 5-MeU phases when compared to respective phases of the LVH group as shown in Fig 3B (S2 Fig).

#### Methoxamine

There was a reduced RCBP responsiveness in the LVH group to ME by 29% in saline phase, 31% in low dose and 43% in high dose 5-MeU phases when compared to same phases of Control group of rats. Treatment of LVH with L-arginine resulted in augmented responses to ME by 110% in the saline, 91% in low dose phase and 217% in high dose 5-MeU phases (P<0.05) when compared to the respective phases in the LVH group as shown in Fig 3C (S3 Fig).

### Renal vasoconstrictor responses of α_1B_–adrenorecptors to adrenergic agonists

#### Nordrenaline

**Fig 4:**
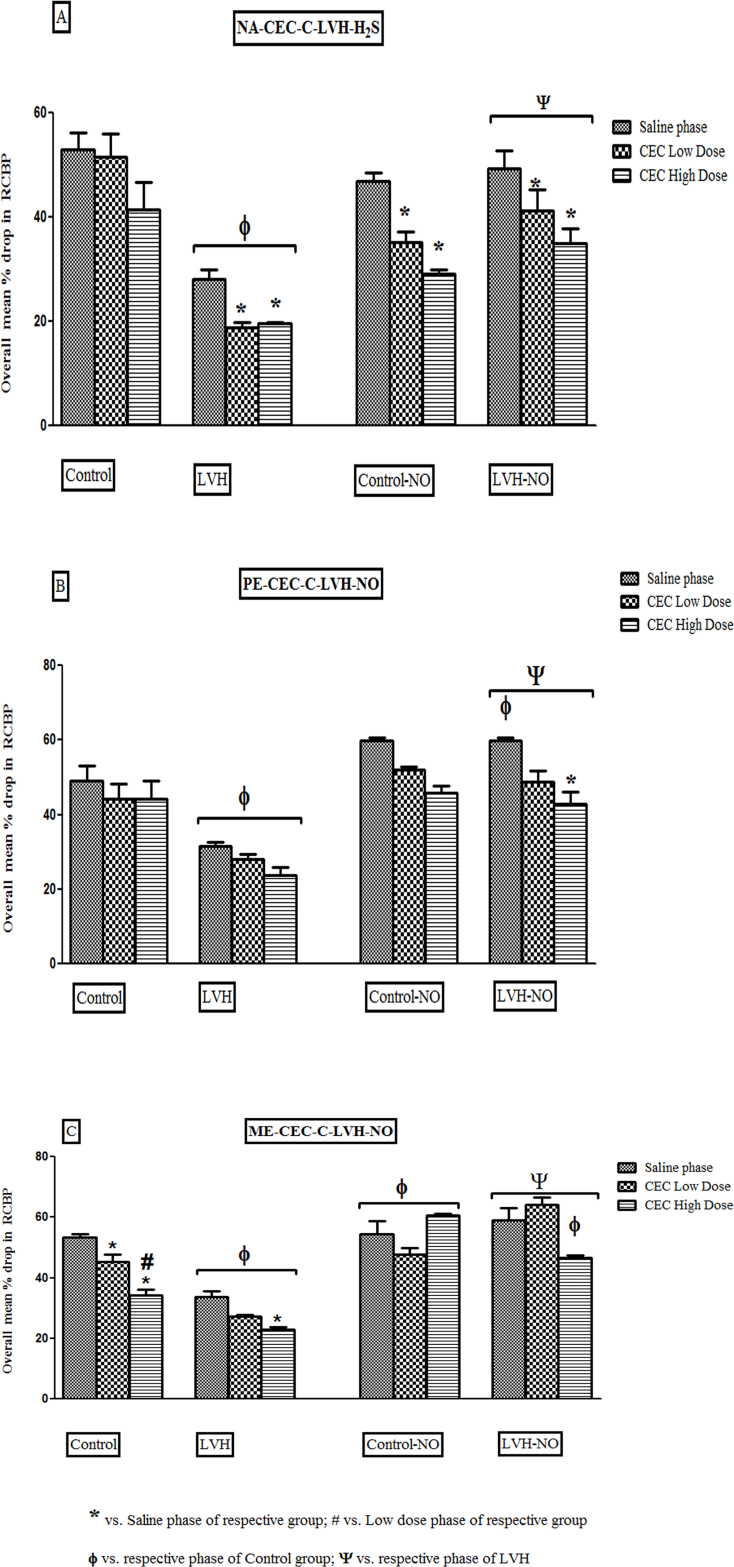
Showing the renal vasoconstrictor responses of α_1B_adrenoreceptors to NA (4A), PE (4B) and ME (4C) in the kidney of Control, LVH, Control-NO and LVH-NO rats. Data is shown as ± SEM while significance is taken as p<0.05.

The responsiveness of RCBP to NA was lower by 47% in the saline, 63% in low dose and 52% in high dose CEC phases when compared to the same phases in the Control groups of rats. Treatment of LVH with L-arginine augmented the RCBP responses to NA by 75% in the saline, 116% in the low dose and 75% in the high dose CEC phase compared to respective phases of the LVH group as shown in Fig 4A (S4 Fig).

#### Phenylephrine

The RCBP responsiveness to PE was lower by 35% in the saline phase, 36% in low dose and 45% in high dose CEC phases compared to same phases in the Control groups of rats. Treatment of LVH with L-arginine resulted in augmented RCBP responses PE by 88% in the saline, 75% in low dose and 79% in the high dose CEC phases compared to the respective phases of in the LVH group as shown in Fig 4B (S5 Fig).

#### Methoxamine

There was a blunted RCBP responsiveness to ME by 36% in the saline, 40% in the low dose and 32% in high dose CEC phases when compared to the same phases in the Control groups of rats. Treatment of LVH with L-arginine resulted in augmented RCBP responses to ME, by 74% in the saline, 137% in low dose % in high dose CEC phases compared to the respective phases in the LVH group as shown in Fig 4C (S6 Fig).

### Renal vasoconstrictor responses of α_1D_–adrenorecptors to adrenergic agonists

#### Nordrenaline

**Fig 5:**
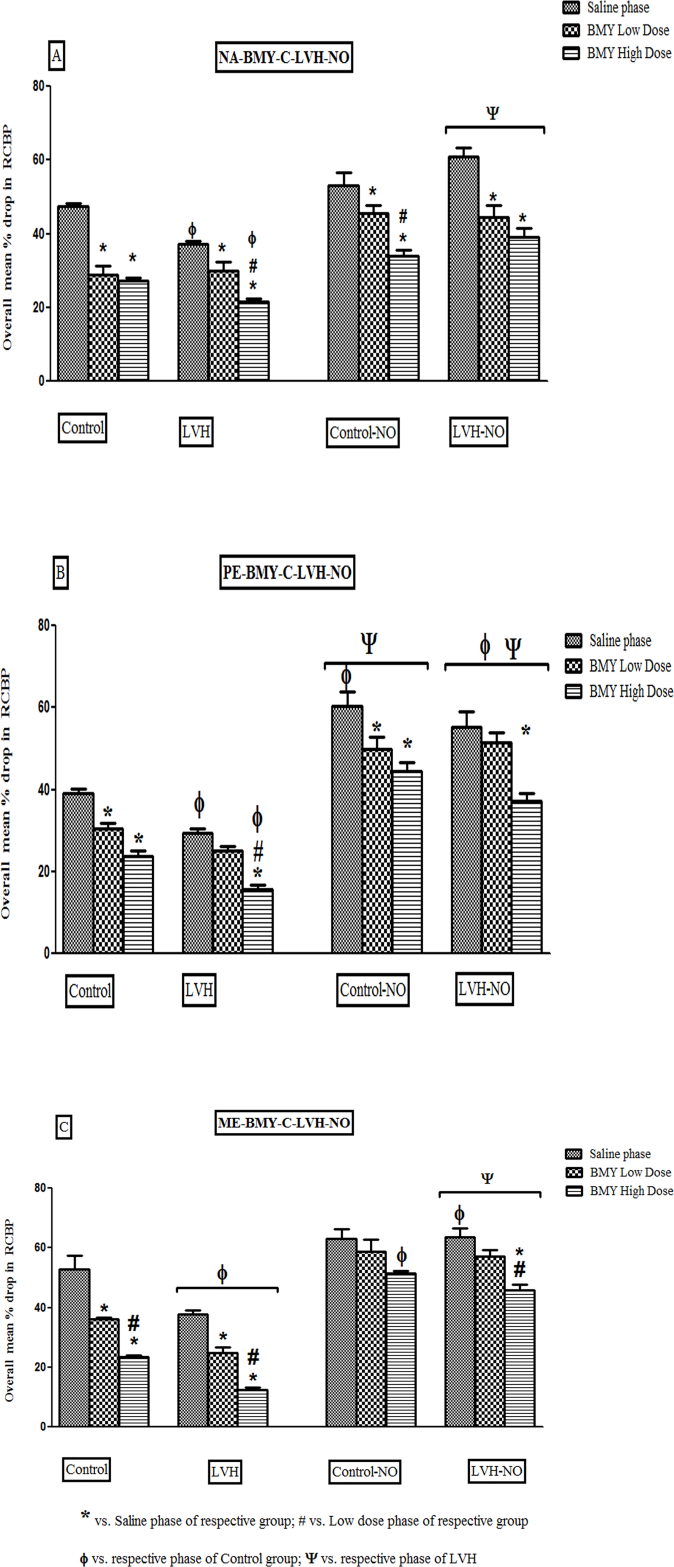
Showing the overall % drop in renal vasoconstrictor responses of α_1D_ adrenoreceptors to NA (5A), PE (5B) and ME (5C) in the kidney of Control, LVH, Control-NO and LVH-NO rats. Data is shown as ± SEM while significance is taken as p<0.05.

In the LVH the RCBP responses to NA were lower by 22% in saline and 19% in high dose BMY phase compared to same phases of Control groups of rats. Treatment of the LVH group with L-arginine augmented the RCBP responses to NA by 65% in the saline, 50% in the low dose and 77% in the high dose BMY phases of when compared to respective phases of the LVH group as shown in Fig 5A (S7 Fig).

#### Phenylephrine

Following LVH induction, there were smaller RCBP responses to PE, by 23% in the saline and 33% in the high dose BMY phase compared to the same phases in the Control group of rats. Treatment of LVH with L-arginine augmented the RCBP responses to PE, by 83% in the saline phase, 104% in low dose BMY phase and131% in the high dose BMY phase compared to respective phases in the LVH group as shown in Fig 5B (S8 Fig).

#### Methoxamine

There was a reduced RCBP responsiveness in the LVH group to ME, by 26% in saline phase and 46% in high dose BMY phase compared to same phases of Control group of rats. Treatment of LVH with L-arginine resulted in augmented RCBP responses to ME, by 68% in the saline phase, 128% in low dose BMY phase and254% in the high dose BMY phase compared to the respective phases in the LVH group as shown in Fig 5C (S3 Fig).

### Histopathological evidence

**Fig 6:**
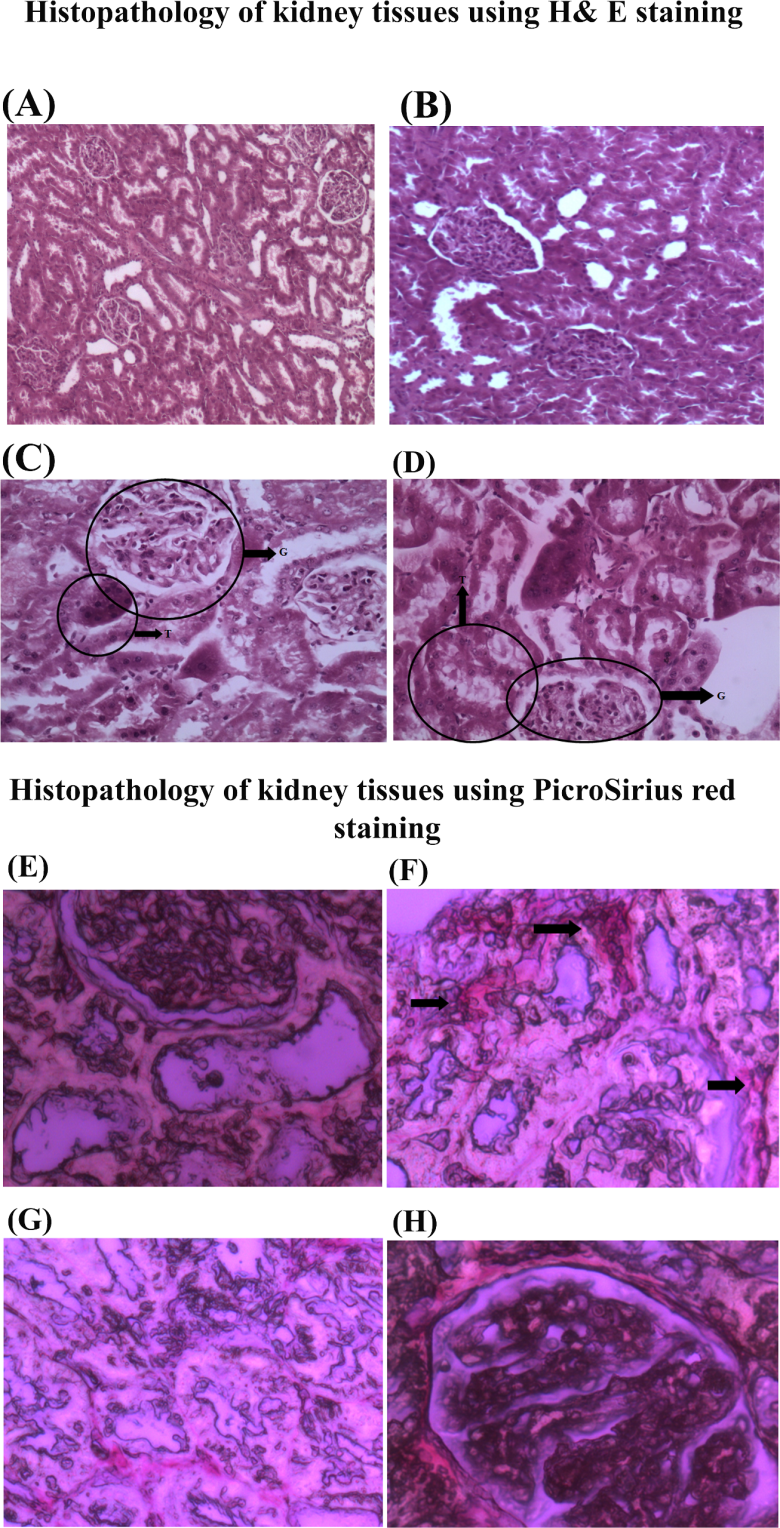
Histopathological study of kidney tissue of Control (A), LVH-WKY (B), Control-NO (C) and LVH-NO (D) for H& E staining while Control (E), LVH-WKY (F), Control-NO (G) and LVH-NO (H) for PicroSirius red staining. Fig 6 shows G and T which represents changes in glomerulus and tubules shape in treatment groups while black arrows pointing out the area of the kidney with collagen plaques.

The kidney tissue did not show any ultra-structural changes in glomerular and tubular components in the LVH group except hypercellularity of the glomerulus with increased mesangial and endothelial cells as shown in Fig 6A and 6B. Treatment with L-arginine in the LVH group resulted in normal glomerular structures but with a mild atrophy of the tubules. Blood vessels and parenchyma were normal in LVH-NO as shown in Fig 6C and 6D).

The kidney tissues of all four groups were subjected to PicroSirius red staining and observed under the polarized light. The collagen content appeared as a red colour as shown in Fig 6E, 6F and 6H) and collagen in the kidney tissue was quantified by using collagen detection software (Image+Pro+Plus6.0, ipwin32, USA).Induction of LVH increased the collagen content, which appeared as plaques around the glomerulus when compared to Control-WKY, as shown in Fig 6E and 6F. However, treatment with L-arginine diffused the bands and collagen deposition was reduced to thin threads and appeared as a network around the glomerulus when compared to the LVH- group as shown in Fig 6G and 6H. Software quantification score showed collagen deposition in Control, LVH, Control-NO and LVH-NO as 0.6%, 3.8%, 1.4% and 1.6% respectively.

## Discussion

The present study was designed to explore the effect of exogenous administration of an NO precursor (L-arginine) on the eNOS/NO/cGMP pathway in the kidney of normal and LVH rats and to investigate whether there was a negative modulatory effect on the CSE/H_2_S pathway in the LVH rats. The major hypothesis was that upregulation of the renal eNOS/NO/cGMP pathway in the kidney would not only prevent the reduced responsiveness in renal cortical blood perfusion but would also tend to normalise the blunted RCBP responses of α_1_-adrenoreceptorsto adrenergic agonists the LVH rats.

The findings clearly showed that there was a down regulation of eNOS mRNA in the cortex of the kidney of the rats given isoprenaline/caffeine to induce LVH and indeed, the NO concentration in the renal cortex was lower. A reduced concentration of NO due to decreased expression of eNOS has previously been reported in the pathological states of hypertension [54] and LVH [19]. There are a number of possible causes for the reduced NO concentration, including reduced NO elaboration from eNOS, increased oxidative inactivation of NO and increased production of vasoconstrictors like endothelin-1 and thromboxane A2 [55, 56]. The down regulation of eNOS/NO occurs at a time when there is increased production of the vasoconstrictors noradrenaline and angiotensin II [3, 6,9] in this model of LVH. Nitric oxide induced vasodilation is followed by an increase in cGMP produced by soluble guanylyl cyclase [57] and in the present study this NO-cGMP axis is attenuated as found in other models of cardiac hypertrophy [58]. Although the down regulation of the eNOS/NO/cGMP was found in the kidney compartment, the fact that there was also reduced plasma NO concentration suggested that a similar situation pertained globally. This down regulation of the renal eNOS/NO/cGMP pathway in the LVH rats was associated with an increased renal vascular tone possibly due to the vasoconstrictor actions of noradrenaline and angiotensin II in the kidney. This would be supported by reports that Ang II suppressed NO-cGMP production [59] and thus elevated production of these vasoconstrictors might be responsible for the reduced basal renal cortical blood pressure in the present study. An attempt was made to provide further evidence for this view by up regulating the eNOS/NO/cGMP pathway using exogenous administration of L-arginine as an NO donor, with the aim of counteracting the effect produced by vasoconstrictors. It was evident that this approach resulted in an increased RCBP, as shown in Figs 2 and 3. Although previous studies have shown that NO increased papillary blood perfusion [15], the present study demonstrated that this also occurred in the renal cortex as renal cortical blood perfusion was increased at a time when the eNOS/NO/cGMP pathway was up regulated in the cortex of the kidney. This would suggest that the buffering action of NO would to a degree offset the actions of vasoconstrictors. Thus, the buffering action of NO to angiotensin II enhanced distensibility and increased eNOS/NO/cGMP pathway fit which would contribute to the increased RCBP.

The responsiveness of α_1A_-adrenoreceptors to adrenergic agonists was attenuated, but not blocked, in the LVH model which indicated that the adrenergically mediated renal vasoconstriction was via α_1A_-adrenoreceptors which are the predominant subtype in renal resistance vessels [60]. This decrease in α_1A_-adrenoreceptor responsiveness may be related to compensatory mechanisms whereby the enhanced sympathetic nervous system activity leads to a down regulation or desensitization of receptors [61]. Sympathetic nervous activity is elevated in this model of LVH as reflected by the increased circulating levels of noradrenaline [3, 6]. It should be pointed out that an increased renal sympathetic nerve activity will also stimulate renin release and hence raise circulating angiotensin II concentrations. An elevation in both noradrenaline and angiotensin II plasma levels could be responsible for the reduced responsiveness of α_1A_-adrenoreceptors which is similar to that previously reported in a fructose fed rat model of LVH [62]. An elevated angiotensin II has been found to be responsible for the suppression of NO-cGMP production [59] as observed in the present study and in cardiac hypertrophy [58]. In order to provide further support for the role of NO in determining the responsiveness of α_1A_-adrenoreceptors to adrenergic agonists, rats were provided with exogenous L-arginine (NO donor) to enhance the eNOS/NO/cGMP signalling cascade. In the LVH this resulted in heightened vasoconstrictor responses to the α_1A_agonists NA, PE and ME even when the receptors were blocked when compared to the same phases in the LVH group as shown in Fig 3A, 3B and 3C). These heightened responses were accompanied at the same time by an up regulation of the eNOS/NO/cGMP pathway in the renal cortex. These findings established an association between the up regulated eNOS/NO/cGMP pathway in the renal cortex and heightened responsiveness of α_1A_-adrenoreceptors to adrenergic agonists in LVH rats treated with L-arginine.

The administration of both the low and high doses of CEC, an α_1B v_adrenoreceptor antagonist, had no effect on the renal vasoconstrictor responses to NA and PE in the Control rats. This was taken to indicate that α_1B_ adrenoreceptors were not functionally contributing to the renal vasoconstriction. These observations support previous studies [45, 63, 64] which found that in normal rats with no renal impairment, there was no functional contribution of α_1B_ adrenoreceptor in mediating the adrenergically induced renal vasoconstriction. However, there were blunted renal vasoconstrictor responses to ME in the Control rats following both the low and high dose CEC phases. This pattern of non-responsiveness to NA and PE but not ME suggested a functional shift of adrenergic receptors with renal vasoconstriction in control rats being mediated by either α_1A_ or α_1D_ adrenoreceptors. The observation that there was reduced renal cGMP levels in the LVH rats suggested an alteration in the G-protein 2^nd^ messenger pathway utilized by α_1_ adrenoreceptors which could be responsible, in part, for reduced responsiveness of these receptors. This view was supported by showing that exogenous administration of NO improved renal cGMP levels in the LVH rats consistent with an upregulation of one of the components of this G-protein 2^nd^ messenger pathway which would contribute to the augmented responsiveness of α_1B_ adrenoreceptor to NA, PE and ME in both basal states and following CEC. It is noteworthy that the functional responses of α_1B_ adrenoreceptors to NA, PE and ME in the LVH-NO group were increased in all three phases when compared to same phases of LVH.

It was evident that in the LVH, the responsiveness of α_1D_ adrenoreceptor activation by NA, PE and ME was blunted in the presence of BMY7378. There was a decrease in the magnitude of responses but they were not completely blocked which indicated a functional contribution of α_1D_ adrenoreceptors in LVH. In the present study there were 65%, 50% and 77% increases in the renal vasoconstrictor responsiveness to NA in the saline phase, low and high dose phases of BMY in the LVH-NO compared to the LVH which strengthens the hypothesis of increased responsiveness of α_1_ adrenoreceptor subtypes in LVH after treatment with L-arginine. The augmented responses of α_1D_ adrenoreceptor activation to adrenergic agonists in the LVH-NO following blockade with BMY7378 indicated a functional involvement and increased responsiveness of this α_1D_ adrenoreceptor subtype in LVH-NO. As PE is a non-selective agonist for all α_1_adrenoreceptor subtypes, the fact that in the exogenous administration of PE in LVH-NO resulted in increased responsiveness to α_1D_ adrenoreceptor activation, by 104% and 131% in the low and high dose BMY7387 phases, respectively, compared to those obtained in the LVH indicated an enhanced involvement of the α_1D_ adrenoreceptor subtype. However, administration of the more selective agonist of α_1D_ adrenoreceptor, ME in the LVH-NO group resulted in augmented responses, of 128% and 254% following blockade with BMY7378 which indicated that the functional contribution of this adrenergic receptor subtype was elevated under these conditions.

The exact mechanism by which exogenous administration of L-arginine increased the responsiveness of α_1_adrenoreceptors in LVH is not clear but it is likely to be of multifactorial origin. There is a view arising from a number of other reports that there is an increased contribution from spare receptors [65, 66].An alternative suggestion, arising from the findings is that exogenous administration of NO donor in LVH up regulated the α_1_-adrenoreceptors whereas in pathophysiological states associated with prolonged hyper sympathetic activity, α_1_-adrenoreceptors have been reported to be down regulated [67] mostly in the renal vasculature [68]. Inhibitors of NO increased renal sympathetic nerve activity [69], the observations of the present study support the concept of a decreased renal sympathetic activity following exogenous administration of NO which could be responsible for the enhanced responsiveness of α_1_-adrenoreceptors to the adrenergic agonists in LVH-NO. A limitation of the present study was that the expression of α_1_-adrenoreceptors in the kidney was not determined. Nonetheless, the sensitivity of these receptors was decreased at a time of elevated renal sympathetic activity in the LVH rats.

More promising evidence for an increased responsiveness was the observations of the modulation of the eNOS/NO/cGMP pathway which is part of G-protein coupled receptor 2^nd^ messenger pathway system. This system was down regulated in LVH but up regulated following elevation of the signalling cascade with L-arginine which demonstrated a clear association between the responsiveness of α_1_-adrenoreceptors and the level of expression of the eNOS/NO/cGMP pathway. L-arginine and α_1_-adrenoreceptors acts through G-protein pathway so it was assumed that upregulation of cGMP pathway is expected to upregulate or increase the responsiveness of the α_1_-adrenoreceptors which are desensitized in LVH and reason for the selection of L-arginine in this study.

It was apparent that there was an interaction between H_2_S and NO as there was a negative impact of the NO donor on renal CSE mRNA expression in LVH rats. These findings contrast with previous reports [27, 70] which concluded that NO was essential for H_2_S production but they are consistent with the suggestion that in normal circumstances where an NO donor enhances plasma concentrations of H_2_S but has an insignificant impact on renal expression of CSE mRNA. This increased H_2_S production in plasma may be due to other H_2_S producing enzymes like *cystathione beta synthase* (CBS). It is possible to conclude from the present findings that there is an interaction between CSE/H_2_S and eNOS/NO under normal conditions but it is abolished in the kidney in LVH. This point of contention is in line with previously reported study [71].

## Conclusion

In summary, the present study explored whether there was a down regulation of eNOS/NO/cGMP pathway in the kidney of LVH rats. It was found that exogenous administration of a NO precursor (L-arginine) in LVH not only increased the renal cortical blood perfusion but also enhanced the blunted responsiveness of α_1_-adrenoreceptors subtypes to adrenergic agonists by up regulating the eNOS/NO/cGMP pathway in the kidney. We also explored whether there was an interaction between the CSE/H_2_S and ENOS/NO cascades under normal conditions but it became apparent that this mutual interaction was abolished in the kidneys of rats with LVH.

## Supporting information

S1 Fig Effects of NA on the responsiveness of α_1A_–adrenorecptors to adrenoreceptor in Control, LVH, Control-NO and LVH-NO groups. * P<0.05 vs. Saline phase; # P<0.05 vs. Low dose MeU.
(DOC)

S2 Fig Effects of PE on the responsiveness of α_1A_–adrenorecptors to adrenoreceptor in Control, LVH, Control-NO and LVH-NO groups. * P<0.05 vs. Saline phase; # P<0.05 vs. Low dose MeU.
(DOC)

S3 Fig Effects of ME on the responsiveness of α_1A_–adrenorecptors to adrenoreceptor in Control, LVH, Control-NO and LVH-NO groups. * P<0.05 vs. Saline phase; # P<0.05 vs. Low dose MeU.
(DOC)

S4 Fig Effects of NA on the responsiveness of α_1B_–adrenorecptors to adrenoreceptor in Control, LVH, Control-NO and LVH-NO groups. * P<0.05 vs. Saline phase; # P<0.05 vs. Low dose CEC.
(DOC)

S5 Fig Effects of PE on the responsiveness of α_1B_–adrenorecptors to adrenoreceptor in Control, LVH, Control-NO and LVH-NO groups. * P<0.05 vs. Saline phase; # P<0.05 vs. Low dose CEC.
(DOC)

S6 Fig Effects of ME on the responsiveness of α_1B_–adrenorecptors to adrenoreceptor in Control, LVH, Control-NO and LVH-NO groups. * P<0.05 vs. Saline phase; # P<0.05 vs. Low dose CEC.
(DOC)

S7 Fig Effects of NA on the responsiveness of α_1D_–adrenorecptors to adrenoreceptor in Control, LVH, Control-NO and LVH-NO groups. * P<0.05 vs. Saline phase; # P<0.05 vs. Low dose BMY.
(DOC)

S8 Fig Effects of PE on the responsiveness of α_1D_–adrenorecptors to adrenoreceptor in Control, LVH, Control-NO and LVH-NO groups. * P<0.05 vs. Saline phase; # P<0.05 vs. Low dose BMY.
(DOC)

S9 Fig Effects of ME on the responsiveness of α_1D_–adrenorecptors to adrenoreceptor in Control, LVH, Control-NO and LVH-NO groups. * P<0.05 vs. Saline phase; # P<0.05 vs. Low dose BMY.
(DOC)

S1 Table Heart index, LV index, R-amplitude and QRS complex of Control WKY, LVH-WKY, Control-WKY and LVH-WKY groups. Heart index, LV index, R-amplitude and QRS complex of Control WKY, LVH-WKY, Control-WKY and LVH-WKY groups on days 35. The values are mean±SEM (n = 6).P<0.05.Statistical analysis was done by one-way analysis of variance followed by Bonferroni *post hoc* test for all the groups. * vs. Control WKY D-35; **#** vs. LVH-WKY D-35.
(DOC)
