# Supplementary material for: Interaction between nitric oxide and renal α1-adrenoreceptors mediated vasoconstriction in rats with left ventricular hypertrophyin Wistar Kyoto rats
Source: PLoS One. 2018 Feb 15;13(2):e0189386. doi: 10.1371/journal.pone.0189386 (PMC5844246; doi:10.1371/journal.pone.0189386)
Supplement: S1 Table — Heart index, LV index, R-amplitude and QRS complex of Control WKY, LVH-WKY, Control-WKY and LVH-WKY groups on days 35. The values are mean±SEM (n = 6).P<0.05.Statistical analysis was done by one-way analysis of variance followed by Bonferroni post hoc test for all the groups. * vs. Control WKY D-35; # vs. LVH-WKY D-35. (DOC) [file pone.0189386.s010.doc]

**S1 Table**

Heart index, LV index , R-amplitude and QRS complex of Control WKY, LVH-WKY, Control-WKY and LVH-WKY groups

| **Groups** | Heart index (%) | LV index (%) | R-amplitude (mV) | QRS (sec) |
| --- | --- | --- | --- | --- |
| Control WKY | 0.26±0.00 | 0.15± 0.00 | 0.53±0.01 | 0.017±0.0001 |
| LVH-WKY | 0.38±0.00 * | 0.23±0.00 * | 0.70±0.02 * | 0.023±0.002 * |
| Control-NO | 0.24±0.00 * | 0.17±0.00 * | 0.61±0.03 | 0.017±0.0005 |
| LVH-NO | 0.30±0.00 # | 0.21±0.00 * # | 0.50±0.01 # | 0.017±0.0002 # |

Heart index, LV index , R-amplitude and QRS complex of Control WKY, LVH-WKY, Control-WKY and LVH-WKY groups on days 35. The values are mean±SEM (n= 6).P<0.05.Statistical analysis was done by one-way analysis of variance followed by Bonferroni *post hoc* test for all the groups ⃰ vs. Control WKY D-35;  **#** vs. LVH-WKY D-35
