# Supplementary material for: Interaction between nitric oxide and renal α1-adrenoreceptors mediated vasoconstriction in rats with left ventricular hypertrophyin Wistar Kyoto rats
Source: PLoS One. 2018 Feb 15;13(2):e0189386. doi: 10.1371/journal.pone.0189386 (PMC5844246; doi:10.1371/journal.pone.0189386)
Supplement: Supplementary file 3 [file pone.0189386.s002.doc]

S2 Fig.

Effect of PE on the responsiveness of α_{1A}-adrenoreceptor in Control, LVH, Control-NO and LVH-NO groups

B

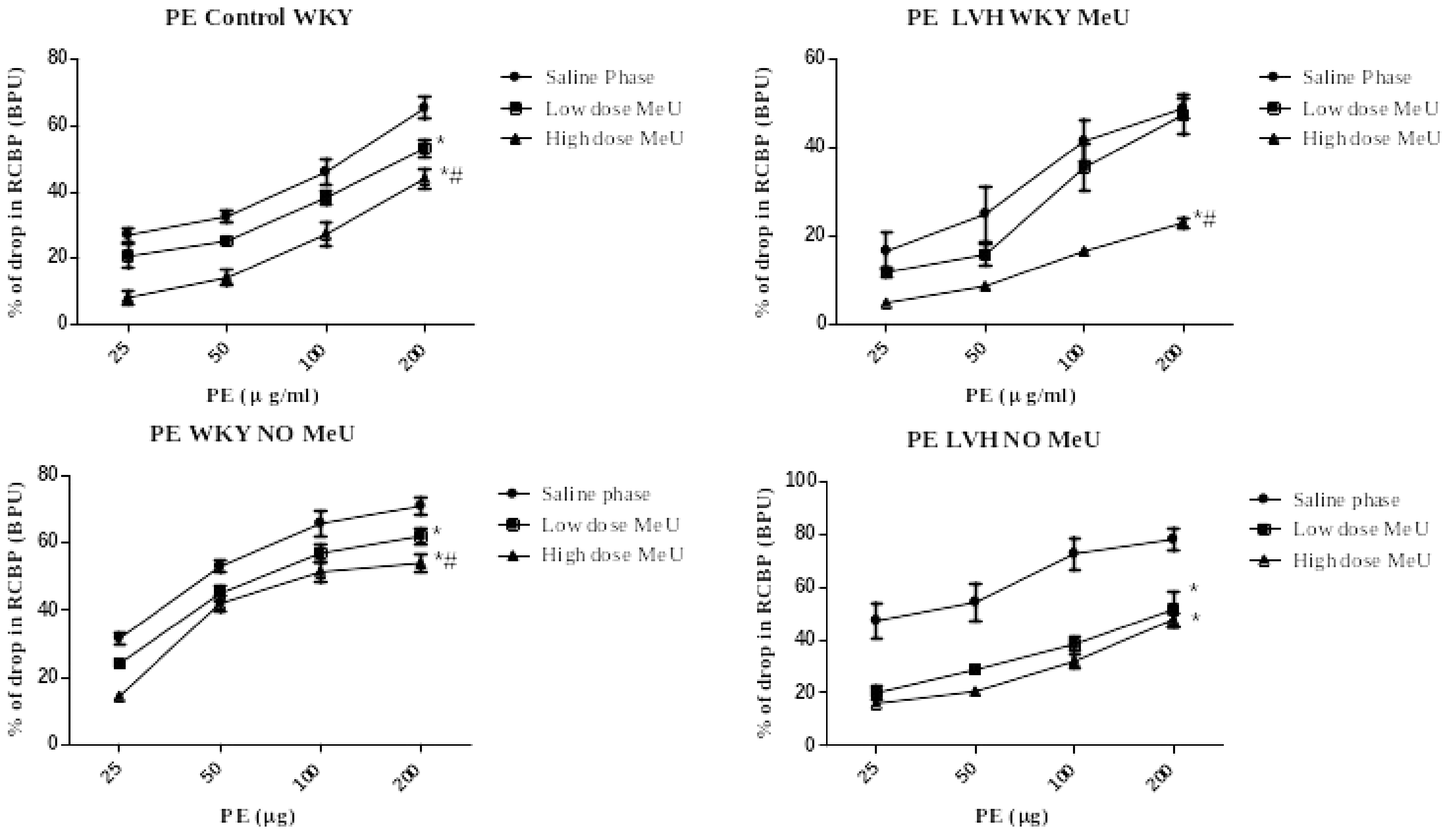

* $P<0.05$ vs. Saline phase; # $P<0.05$ vs. Low dose MeU
