# Supplementary material for: Interaction between nitric oxide and renal α1-adrenoreceptors mediated vasoconstriction in rats with left ventricular hypertrophyin Wistar Kyoto rats
Source: PLoS One. 2018 Feb 15;13(2):e0189386. doi: 10.1371/journal.pone.0189386 (PMC5844246; doi:10.1371/journal.pone.0189386)
Supplement: Supplementary file 5 [file pone.0189386.s004.doc]

# S4 Fig.

## Effect of NA on the responsiveness of $\alpha_{1B}$-adrenoreceptor in Control, LVH, Control-NO and LVH-NO groups

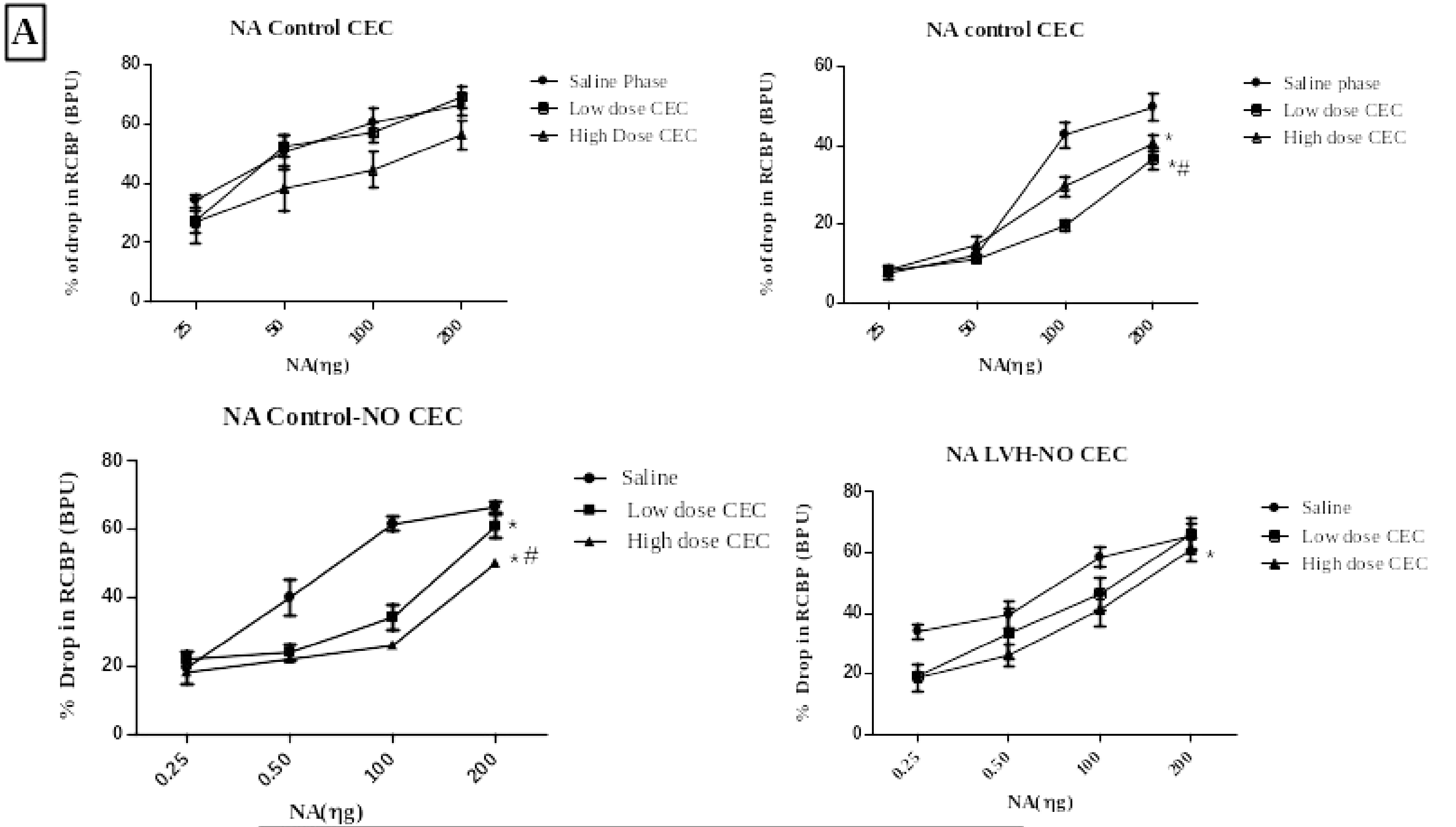


* P<0.05 vs. Saline phase; # P<0.05 vs. Low dose CEC
