# Supplementary material for: Interaction between nitric oxide and renal α1-adrenoreceptors mediated vasoconstriction in rats with left ventricular hypertrophyin Wistar Kyoto rats
Source: PLoS One. 2018 Feb 15;13(2):e0189386. doi: 10.1371/journal.pone.0189386 (PMC5844246; doi:10.1371/journal.pone.0189386)
Supplement: Supplementary file 6 [file pone.0189386.s005.doc]

S5 Fig.

Effect of PE on the responsiveness of α_{1B}-adrenoreceptor in Control, LVH, Control-NO and LVH-NO groups

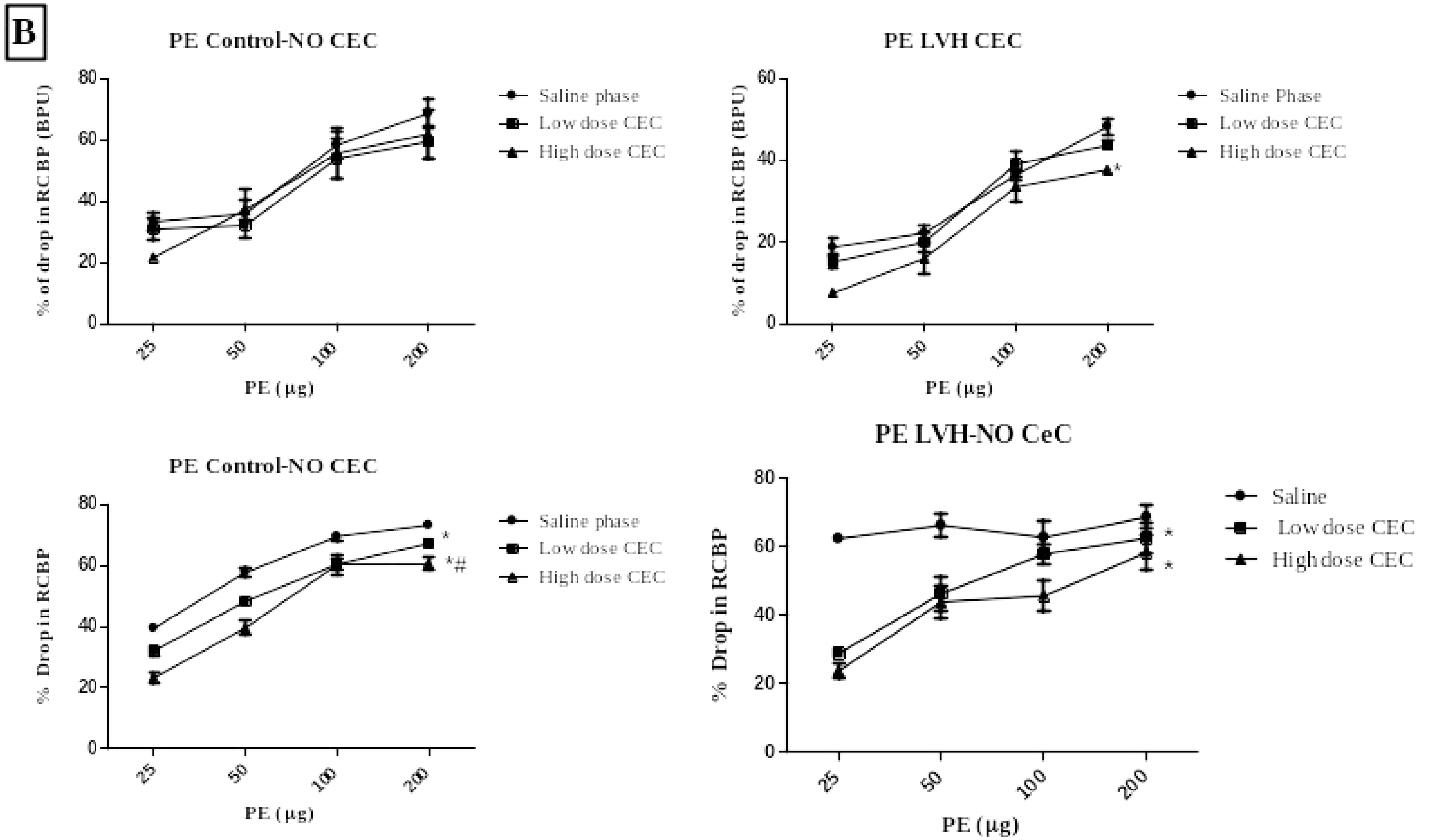

* P<0.05 vs. Saline phase; # P<0.05 vs. Low dose CEC
